# Understanding the impact of COVID-19 on dental antibiotic prescribing across England: 'it was a minefield'

**DOI:** 10.1038/s41415-022-5104-y

**Published:** 2022-10-28

**Authors:** Wendy Thompson, Sagar Shah, Valerie Wordley, David Edwards

**Affiliations:** 4141519928001 Division of Dentistry, University of Manchester, Manchester, M13 9PL, UK; 4141519928002 Acute Dentistry, Cardiff and Vale University Health Board, Cardiff, CF14 4XW, UK; 4141519928003 NHS England, London, SE1 6LH, UK; 4141519928004 School of Dental Sciences, Newcastle University, Newcastle-upon-Tyne, NE2 4BW, UK

## Abstract

**Introduction** The COVID-19 pandemic impacted significantly on dental service delivery across England.

**Objective **To explore how and why the pandemic affected antibiotic prescribing by primary care dentists.

**Methods** Mixed-methods study: secondary analysis of routinely collected NHS dental antibiotic prescribing data from before and during the pandemic; and an online survey of dentists (NHS and private) across England's regions.

**Results ** Dental antibiotic prescribing increased 22% in the pandemic's first year, with regional variation from a 12.1% increase in London to 29.1% in East of England. Of 159 dentists surveyed, 60% had prescribed 'many more' antibiotics. Some urgent dental centres required antibiotics before accepting referrals, irrespective of clinical appropriateness. Diagnosing remotely was hard. Antibiotics were used to delay aerosol generating procedures and fill gaps in services. Widespread frustration existed.

**Discussion** Maintaining access to urgent dental care during a future pandemic would include availability of high-grade personal protective equipment for dental teams. Everyone must understand the impact of restricted dental access on antibiotic overprescribing and hence antibiotic resistance.

**Conclusion** Reduced dental access and changes in dental service delivery because of the pandemic increased antibiotic prescribing. Ensuring uninterrupted access for all to effective urgent dental care is an important element of global efforts to tackle antibiotic resistance.

**Supplementary Information:**

Zusatzmaterial online: Zu diesem Beitrag sind unter 10.1038/s41415-022-5104-y für autorisierte Leser zusätzliche Dateien abrufbar.

## Key points

- Remote management of patients during the COVID-19 pandemic resulted in increased antibiotic prescribing in each of England's regions. Most dentists were not confident managing patients with acute dental conditions remotely. Ensuring access to face-to-face dentistry is an important part of efforts to tackle antibiotic resistance.
- Dentists felt frustrated about being prevented from providing the dental procedures needed to provide safe and effective care to people in pain. Many also expressed concern about the impact of increased antibiotic prescribing on antibiotic resistance and their experience of increased patient expectation that antibiotics are appropriate for toothache.
- Preparedness plans for future pandemics should recognise that antibiotics are not a substitute for dental procedures and dentistry was the only part of the NHS to experience an increase in antibiotic prescribing during the COVID-19 pandemic. Planners should aim to ensure sufficient face-to-face urgent dental care provision immediately upon implementation of pandemic restrictions.

**Supplementary Information:**

Zusatzmaterial online: Zu diesem Beitrag sind unter 10.1038/s41415-022-5104-y für autorisierte Leser zusätzliche Dateien abrufbar.

## Introduction

Antibiotic resistance is both a global public health and patient safety problem, driven by the use of antibiotics.^1^ In 2019, antibiotic resistant infections accounted for more deaths than human immunodeficiency virus and malaria combined.^2^ By 2050, an estimated ten million lives a year and a cumulative $100 trillion of economic output are at risk.^3^ As the dental profession contributes significantly to the burden of antibiotic prescribing in England (being responsible for more than 10% across primary healthcare),^4^ it has an important role in slowing the development and spread of antibiotic resistant infections.

Guidelines in the UK are based on the principle that most dental infections are amenable to operative procedures rather than antibiotic prescriptions. Antibiotics are not routinely recommended to prevent local or distant site infections (such as infective endocarditis).^5^ Before the COVID-19 pandemic, high rates of antibiotic prescribing had been described in the UK, of which around 80% were considered unnecessary or inappropriate.^6^

**Fig. 1:**
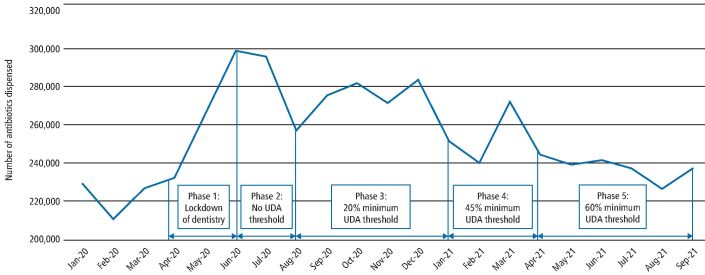
Number of antibiotic items dispensed by community pharmacists related to NHS dental FP10D prescriptions in England per month between January 2020 and September 2021. Highlighted are the key phases in the dental response to the COVID-19 pandemic showing activity (units of dental activity [UDA]) thresholds for full NHS contract payments. Source: NHSBSA dental prescribing data, NHSBSA Copyright 2022 is licenced under the terms of the Open Government Licence

The COVID-19 pandemic has had a significant impact on dentistry in England,^7^ from a complete closure of all NHS and private dental practices (23 March to 7 June 2020)^8^ followed by a staged restoration of dental services in line with the easing of COVID-related safety measures (see Figure 1).^9^ During the period of lockdown, dental practices were instructed to stop seeing patients face to face and to manage patients with acute dental problems remotely with advice, analgesics, or antimicrobials (AAA), where appropriate. Only designated NHS urgent dental centres (UDCs) could provide dental procedures on referral from other services.^8^

Dentistry was the only part of the NHS in England which experienced an increase in antibiotic prescribing during the first year of the COVID-19 pandemic.^10^ This study aimed to describe that increase and to explore dentists' perspectives about why it occurred.

## Methods

A mixed-methods study consisting of two parts: i) secondary analysis of routinely collected NHS prescribing data; and ii) a survey of dentists (NHS and private) working across England since at least April 2019 (before COVID-19).

### Analysis of NHS prescribing data

The total number of antibiotic items (as per British National Formulary section 5.1: 'antibacterial drugs') dispensed by community pharmacists in England relating to NHS dental prescription forms (FP10D) were sourced from the NHS Business Services Authority.^11^ Monthly totals for each of NHS England's seven regions were provided for the period January 2019 to September 2021. Prescriptions dispensed from prisons, hospitals and private prescriptions were not included.

To enable comparisons between regions, the rate of prescriptions dispensed per 1,000 of the population were calculated. Mid-year population estimates for 2019 and 2020 were obtained from the Office for National Statistics.^12^ In the absence of more up-to-date figures, population estimates for 2020 were used to calculate the 2021 rates.

As these datasets were publicly available and completely anonymised, ethical approval was not required (NHSBSA Data Warehouse, NHSBSA Copyright 2022. This information is licenced under the terms of the Open Government Licence).^11^

### Survey of dentists

An online survey of dentists working in primary dental care settings in England since March 2019 was undertaken using the Qualtrics XM tool. Participants were recruited via the online Facebook group 'For dentists by dentists' during the three-month period July to September 2021. The sample size of 136 responses was calculated by assuming that at least 90% of dentists would have increased their antibiotic prescribing since the start of the COVID-19 pandemic compared to the previous 12 months (the primary research question). Dentists who had not practised clinical dentistry in primary dental care in England since March 2019 were excluded.

The survey consisted of 20 questions (see online Supplementary Information), with a mixture of multi-choice and free-text answers and was piloted with ten dentists from across NHS England's regions. Data were analysed using descriptive and inferential statistics to explore factors which may have affected dental antibiotic prescribing during the pandemic.

Ethical approvalfor the survey was gained from University of Manchester UREC Ref. 2021-12282-19832 dated 29/06/2021. All participants consented to participate in the study and to have their data used as part of the research.

## Results

### Analysis of NHS prescribing data

A 22% increase in the number of NHS dental antibiotic prescriptions occurred in the first year of COVID-19 restrictions (3,230,225 items between April 2020 and March 2021) compared to the previous year (2,642,391 items between April 2019 to March 2020). The greatest increase occurred in the first phase when dental practices were closed (March to June 2020) (Fig. 1). Since the reopening of dental practices in June 2020, there has been a downward trend of antibiotic prescribing.

**Fig. 2:**
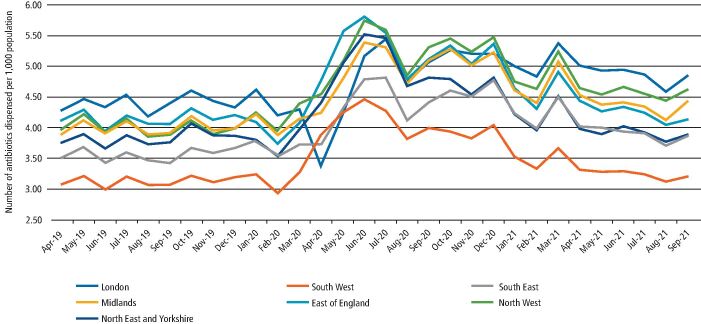
Rate of antibiotic items dispensed by community pharmacists related to NHS dental FP10D prescriptions per 1,000 population in each NHS region of England by month between April 2019 and September 2021. Source: NHSBSA dental prescribing data, NHSBSA Copyright 2022 and Office of National Statistics Population Estimates are licenced under the terms of the Open Government Licence

In the year before COVID-19 pandemic restrictions, all regions experienced reducing rates of dental antibiotic prescribing (see Figure 2). After the introduction of COVID-19 restrictions (March 2020), most areas experienced a steep increase (April and May) until practices reopened (June 2020). However, London experienced a markedly different pattern, with a large reduction in April followed by a steeper rise than the other regions in May and June 2020. Overall, the London region experienced a 12.1% rise in dental antibiotic prescribing in 2020-2021 compared to 2019-2020, the smallest increase across the seven regions. The East of England experienced the largest increase (29.1%); more details are in online Supplementary Table 1. After practices reopened in June 2020, all regions experienced a general downward trend in dental antibiotic prescribing.

### Survey of dentists

In total, 159 dentists responded to the survey from across all seven NHS regions. Most were UK dental school graduates (84%) and 58% identified as women (see Table 1).

**Table 1:** Demographics of the survey respondents (n = 159)

Demographic		n (%)
Primary dental qualification	UK dental school	134 (84.2)
	EU dental school	15 (9.4)
	Non-EU dental school	8 (5.0)
	Did not say	2 (1.3)
Region of work since start of pandemic	East of England	19 (11.9)
	London	17 (10.7)
	Midlands	23 (14.5)
	North East and Yorkshire	28 (17.6)
	North-West	26 (16.4)
	South-East	23 (14.5)
	South-West	14 (8.8)
	Unsure/Did not say	9 (5.7)
Worked in UDC system during COVID-19	Yes	28 (17.6)
	No	129 (81.1)
	Did not say	2 (1.3)

#### Patterns of individual prescribing

Overall, 89% (141/159) of dentists reported that their dental antibiotic prescribing had increased in the first year of the pandemic (April 2020 to March 2021) compared to the previous year. Just three dentists reported prescribing antibiotics less often. Most had prescribed antibiotics remotely (n = 128) at least once during the initial two months when practices were closed:

- '*Forcing dental practices to not see patients face to face during the early stages of the pandemic led to a reduction in the level of patient care and an unnecessary increase in the prescribing of antibiotics*'.

#### Alignment of national and local guidelines/protocols

Most respondents felt that they understood the national guidelines for remote management issued on 25 March 2020, although nearly three-quarters (72%; 102/141) reported that local guidelines did not align well with national guidelines. As shown in online Supplementary Table 2, most dentists from each of the regions (except the South West) reported this poor alignment and there was no significant difference between the regions (p = 0.587). The South West was the only region where most (54%; 6/11) felt the local and national guidelines aligned well and generally had the lowest overall rate of dental antibiotic prescribing (see Figure 2).

Common issues reported about this misalignment between local and national guidelines and protocols related to local mandating of antimicrobial prescribing (sometimes dual antimicrobial therapy) before acceptance of a referral by a UDC for treatment, irrespective of the referring dentists' assessment about whether antimicrobials were appropriate:

- '*[Antibiotics] were almost a prerequisite for getting an appointment, even though it doesn't work for pulpitis'*.

Half of the dentists (71/140, 51%) reported that, during the first phase of COVID-19 restrictions (23 March to 7 June 2020), their referrals to a UDC had been rejected because the patient had not first taken antibiotics:

- '*Patients were refused to be seen at an urgent dental centre for treatment until they'd had antibiotics*'
- '*I had to prescribe for a loose tooth as [the UDC] wouldn't see the patient otherwise'*.

In more than one of the regions, dentists reported that dual prescribing of antibiotics had been required:

- '*We were told we had to follow the [local] protocols for prescribing two antibiotics at the same time*'.

Various reasons were proposed for this misalignment between effective care for people with acute dental problems and the practices operated by some UDCs, including insufficient availability of face-to-face urgent dental appointments and mainstreaming of remote prescribing.

- '*We had such limited capacity in our UDC*'
- '*Some areas still do remote prescribing in a UDC. The service is commissioned to do this'*.

#### Confidence diagnosing and treating remotely

Fewer than half (68/140; 49%) of respondents were confident remotely diagnosing and just one-quarter (35/140; 25%) were confident remotely treating patients with acute dental pain or infection. Dentists who had worked in UDCs during the first year of COVID-19 were significantly more confident (p = 0.001) remotely diagnosing patients with acute dental conditions (18/27; 66.7%) than the other dentists (49/112; 43.8%) (see online Supplementary Table 2).

#### Antibiotics to delay definitive dental procedures

Some dentists were concerned that they were unable to provide the dental procedures which are normally indicated for the treatment of toothache. Removal of an inflamed dental pulp (extirpation) was classified in NHS standard operating procedures as an aerosol generating procedure (AGP) and hence was subject to additional infection prevention and control measures in dental practices. Even though antibiotics do not cure this type of toothache, some dentists were nevertheless prescribing antibiotics until a dental procedure could be provided for the patient.

- '*I can no longer [provide] a quick extirpation at an urgent appointment like I would have done and therefore I am prescribing a lot more antibiotics*'
- '*Massive increase in patients being referred in [to the endodontic referral practice] who have had irreversible pulpitis managed with multiple courses of antibiotics (obviously) without success*'.

Delaying AGPs was an enduring problem for some, with 23 respondents reporting it as still being an issue after April 2021.

#### Patient requests, negotiation and expectations

Over three-quarters (109/139, 78%) reported patients requesting antibiotics more often during the first year of the COVID-19 pandemic than the previous year:

- '*Yes more [antibiotics] as patients demanded them*'.

Other dentists felt that patients would report their symptoms in such a way as to negotiate for their preferred treatment outcome:

- '*The difficulty I came across was the inaccurate description of the acute dental pain of the patients in order to get the antibiotics or painkiller*'.

Dentists suggested that remote management of patients (with AAA) during the pandemic had had a lasting effect on patient expectations about the ability to use antibiotics to avoid a dental procedure:

- '*Patients now think they can call for antibiotics when they wouldn't before and they use the fact they have COVID as an excuse as to why they can't come in. (Maybe scepticism but patients who have been told extraction is needed etc)*'.

#### Filling gaps in NHS dental service provision

Several dentists (from more than one NHS region) raised the continued use of remote antibiotic prescribing to fill gaps in NHS dental service provision as an issue not covered by the quantitative questions within the survey:

- '*There are still not enough urgent dental slots within our service and we are still offering antibiotics as the first line treatment*'.

#### Sense of frustration

Running throughout the 'free-text' comments was a sense of frustration. Many dentists felt their autonomy in clinical decision-making was being 'compromised', that they had 'no choice' in clinical decision-making and that were being 'forced' to provide care in a way which they felt was inappropriate:

- '*I feel horribly compromised as I feel this is far from ideal and I worry about antibiotics*'
- '*I felt that the AAA guidelines were totally against all I had been taught about treating dental pain, but I felt that I had no choice but to prescribe antibiotics on many occasions as we were not allowed to treat patients face to face*'
- '*We were forced to prescribe antibiotics remotely*'
- '*I lied a lot. When patients had pulpitis, I told them to say that I'd prescribed antibiotic as a means of being seen at the Hub. Antibiotic would not have been appropriate*'
- '*It was a minefield!*'.

## Discussion

Remote management of patients during the COVID-19 pandemic resulted in dramatic increases in antibiotic prescribing in each of England's regions, with an overall increase of 22% in the first year of the pandemic compared to the year before it. A variety of factors have been identified which may have impacted on dental antibiotic prescribing as a result of the COVID-19 pandemic, including dentists finding it hard to diagnose patients remotely, UDCs requiring antibiotics to have been prescribed irrespective of their clinical appropriateness, the use of antibiotics to delay AGPs, and to fill gaps in NHS dental service provision. Many expressed concerns about the enduring impact of increased antibiotic prescribing during the pandemic on antibiotic resistance and on increased patient expectations about antibiotics being appropriate for toothache and for avoiding dental procedures.

England is not the only country to experience increased dental antibiotic prescribing related to remote management of patients during the pandemic.^7^ Even larger increases have been reported in primary dental care in Scotland (49%)^13^ and in the public health dental clinics of Alberta, Canada (76%).^14^ By contrast, reductions in dental antibiotic prescribing at the start of pandemic restrictions were reported in primary dental care in Australia (20%)^15^ and in Qatar, only 7% of patients managed remotely with teledentistry were prescribed antibiotics.^16^ In France, a similar increase (17%) was experienced in 2020 compared to 2019.^17^ Further research is indicated to understand the reasons for these differences between countries.

The widespread sense of frustration expressed by dentists about their inability to provide effective care for patients with severe toothache reflects issues identified in a survey of Scottish dentists.^11^ A House of Commons (HoC) Health Select Committee (HSC) review on the impact of COVID-19 on the NHS^18^ noted that people were returning in pain as they were being given antibiotics instead of procedures to treat the cause of the problem. The HSC also noted that staff in dental practices need access to 'particular types' of personal protective equipment (PPE), such as filtering facepiece respirators, to maintain the safety of staff and patients. A joint 'lessons learned' report from the HSC and HoC Science and Technology committees about the COVID-19 pandemic recognised that dentistry had been particularly badly affected because of the prevalence of AGPs during routine care.^19^ Ensuring access to face-to-face dentistry for people with toothache is an important part of efforts to deliver the government's vision to keep antibiotics working as effectively as possible for as long as possible and involves seemingly unrelated issues, such as the availability of appropriate PPE for dental teams during a pandemic.

Confusion about the way some local guidelines and protocols did not reflect national guidelines on the management of dental patients with acute dental conditions was a significant concern. Further research is indicated to understand this issue from the perspective of UDCs and other organisations involved with the delivery of dental care during the COVID-19 pandemic. Preparedness plans for national emergencies should ensure clarity about the 'where appropriate' element of any future AAA approach. Training for implementation of emergency plans should also ensure that those responsible for commissioning and managing the delivery of NHS dental services understand the essential role of effective antibiotics for treating life-threatening infection and the futility of antibiotics for toothache such as pulpitis.

Those with experience of working in the UDCs were more confident diagnosing and treating patients with acute dental conditions remotely than others in primary dental care. A systematic review of factors influencing dental antibiotic prescribing found an issue around the level of skill and self-belief of some dentists about providing dental procedures during urgent appointments.^20^ An ethnographic study undertaken shortly before COVID-19 in out-of-hours (OOH) dental clinics and general dental practice found that dentists working OOH tend to be more confident at providing urgent dental care, especially numbing difficult teeth and for anxious patients.^21^ To ensure that the dental workforce is prepared to deal with a future pandemic and to improve access to urgent dental care for the whole population, it is suggested that the future NHS general dental services contract should include a requirement to provide urgent dental care to patients who are not regular attenders at that practice.

Interestingly, in the first month of the pandemic restrictions, the London region experienced a markedly different pattern in the rate of dental antibiotic prescribing compared to the other six regions. This drop in dental antibiotic prescribing by primary care dentists may reflect the existence of multiple dental hospitals in the capital which rapidly became UDC hubs.^22^ This pattern of dental hospitals rapidly transitioning to UDCs was reflected elsewhere in the country,^21^^,^ although most other regions had fewer dental hospitals to serve their populace.^23^^,^^24^ Analysis of care provided for patients before referral into UDC hubs found that half of their patients had received a course of antibiotics before their onward referral into a UDC for treatment, with many appearing to be an inappropriate use of antibiotics.^25^^,^^26^ No differences were apparent in the survey results between the NHS regions, although the relatively low number of participants per region made statistical comparison inappropriate. Future preparedness planners should include evidence from around the UK about the best way to deliver UCDs quickly during a pandemic in areas with and without dental hospitals.

It is important to highlight that this study's quantitative analysis was limited to antibiotic prescribing by NHS dentists and includes neither dental antibiotic prescribing in dental hospitals nor private dental practices, which account for around 25% of primary care dental provision in England. No source of routinely collected data exist for these settings. Given the restricted access to healthcare services experienced during the pandemic, it is also likely that many antibiotics were issued to people with acute dental conditions outside of NHS primary dental care (such as general practitioners or emergency departments). Further research is indicated to understand the magnitude of antibiotic prescribing for acute dental problems in other healthcare settings, to understand reasons for dental attendance in these settings and to identify care pathways so that safe and effective care can be provided for those patients through dental procedures, rather than antibiotic prescriptions.

## Conclusion

Reduced dental access and changes in dental service delivery because of the pandemic increased antibiotic prescribing. Ensuring uninterrupted access for all to high-quality urgent dental care is an important element of global efforts to tackle antibiotic resistance. This includes availability of appropriate PPE for dental teams during a pandemic and training of the wider team about appropriate treatment for acute dental conditions. Understanding the essential role of effective antibiotics for treating life-threatening infections and their futility for many acute dental conditions should be fundamental knowledge for everyone involved with planning, managing and delivering dental services.

## Supplementary Information

Supplementary Information (PDF 165KB)
