# Supplementary material for: Understanding the impact of COVID-19 on dental antibiotic prescribing across England: 'it was a minefield'
Source: Br Dent J. 2022 Oct 28;233(8):653–8. doi: 10.1038/s41415-022-5104-y (PMC9615612; doi:10.1038/s41415-022-5104-y)
Supplement: Supplementary file 1 — Supplementary Information (PDF 165KB) [file 41415_2022_5104_MOESM1_ESM.pdf]

Table S1: Comparing antibiotic prescribing in each region of NHS England in the year before the COVID-19 restriction on dental services (April 2019 to March 2020) and the first year of those restrictions (April 2020 to March 2021). Data source: *NHSBSA dental prescribing data*, NHSBSA Copyright *2022* is licenced under the terms of the Open Government Licence.

	East of England	London	Midlands	North-East & Yorkshire	North-West	South-East	South-West
Apr 2019	25,652	38,346	41,923	30,751	29,130	32,246	17,367
May 2019	26,807	40,023	44,447	31,864	31,032	33,926	18,089
Jun 2019	24,567	38,852	42,093	29,967	28,912	31,501	16,837
Jul 2019	26,265	40,715	44,224	31,758	30,535	33,113	18,051
Aug 2019	25,340	37,444	41,978	30,498	28,286	31,844	17,261
Sep 2019	25,299	39,404	42,172	30,763	28,539	31,470	17,308
Oct 2019	26,961	41,338	45,216	33,348	30,353	33,780	18,186
Nov 2019	25,750	39,809	42,594	31,704	28,608	33,003	17,520
Dec 2019	26,252	38,812	42,976	31,693	29,279	33,717	18,011
Jan 2020	26,869	41,624	45,119	32,906	30,196	33,953	18,412
Feb 2020	24,545	37,838	41,317	30,565	27,966	31,732	16,604
Mar 2020	26,790	38,715	44,268	34,420	31,155	33,332	18,556
**Total 2019-20**	**311,097**	**472,920**	**518,327**	**380,237**	**353,991**	**393,617**	**212,202**
	**East of England**	**London**	**Midlands**	**North-East & Yorkshire**	**North-West**	**South-East**	**South-West**
Apr 2020	31,318	30,325	45,239	38,099	32,245	33,371	22,066
May 2020	36,534	37,863	51,096	43,710	35,933	38,559	24,024
Jun 2020	38,154	46,577	57,485	47,746	40,783	42,828	25,327
Jul 2020	36,436	48,977	56,615	47,150	39,709	43,052	24,265
Aug 2020	31,266	42,602	50,139	40,384	34,332	36,737	21,619
Sep 2020	33,639	45,689	54,657	41,653	37,688	39,480	22,655
Oct 2020	35,064	47,464	56,045	41,477	38,637	41,195	22,333
Nov 2020	33,022	46,853	53,510	39,190	37,115	40,233	21,704
Dec 2020	35,242	46,827	55,796	41,638	38,814	42,632	22,933
Jan 2021	30,427	45,025	48,888	36,324	33,569	37,725	19,916
Feb 2021	28,282	43,515	46,954	34,190	32,838	35,597	18,924
Mar 2021	32,236	48,452	54,267	38,989	37,199	40,342	20,816
**Total 2020-21**	**401,620**	**530,169**	**630,691**	**490,550**	**438,862**	**471,751**	**266,582**
**% Change from 2019 - 20 to 20 -21**	**29.1**	**12.1**	**21.7**	**29.0**	**24.0**	**19.9**	**25.6**

Table S2: Cross tabulation of: a) dentists' experience working in the urgent dental care (UDC) system during the COVID-19 pandemic and b) region of NHS England against i) their confidence treating patients remotely; ii) diagnosing acute dental problems remotely; and iii) their views about how well local guidelines aligned with national guidelines during the pandemic. Pearson Chi-square undertaken with Bonferroni adjustment.

		**How confident did you feel treating acute dental pain or infection remotely?**			**How confident did you feel diagnosing acute dental pain or infection remotely?**			**How well did local guidelines and practices align with national guidance on patient management?**		
		Confident*	Unconfident*		Confident*	Unconfident*		Well**	Less well**	
		n (%)	n (%)	Chi-square	n (%)	n (%)	Chi-square	n (%)	n (%)	Chi-square
Experience working in UDC	Yes	12 (44.4)	15 (55.6)	p=0.01	18 (66.7)	9 (33.3)	p=0.03	11 (40.7)	16 (59.3)	p=0.08
	No	23 (20.7)	88 (79.3)		49 (43.8)	63 (56.3)		27 (23.9)	86 (76.1)	
Region of NHS England	East of England	3(25.0)	9 (75.0)	p=0.70	9 (75.0)	3 (25.0)	p=0.37	3 (23.1)	10 (76.9)	p=0.59
	London	3 (30.0)	7 (70.0)		3 (30.0)	7 (70.0)		4 (40.0)	6 (60.0)	
	Midlands	2 (15.4)	11 (84.6)		5 (38.5)	8 (61.5)		3 (23.1)	10 (76.9)	

	North-East & Yorkshire	5 (20.8)	19 (79.2)		11 (45.8)	13 (54.2)		6 (25.0)	18 (75.0)	
	North-West	3 (15.8)	16 (84.2)		9 (47.4)	10 (52.6)		5 (26.3)	14 (73.7)	
	South-East	7 (38.9)	11 (61.1)		8 (44.4)	10 (55.6)		6 (33.3)	12 (66.7)	
	South-West	3 (27.3)	8 (72.7)		7 (63.6)	4 (36.4)		6 (54.5)	5 (45.5)	

* 'Confident' includes extremely & somewhat confident. 'unconfident' includes extremely or somewhat and neither confident nor unconfident)

** 'Well' includes extremely and very well. 'Less well' includes 'slightly & moderately well'

Survey questions

Q1 Before agreeing to participate with this study, please read the participant information sheet. You can find out more information about how the University handles your data in the privacy policy.

I have read the participant information sheet and consent to take part in this study.

I do not consent to participate.

Q2 Are you a registered dentist who has practised clinical dentistry in primary dental care in England since March 2019 (i.e. for at least a year before the COVID pandemic)?

Yes

No

Q3 What best describes your gender?

Male

Female

In another way

Prefer not to say

Q4 When did you qualify as a dentist?

Before 1970

1970-1979

1980-1989

1990-1999

2000-2009

2010-2019

Q5 Where did you gain your primary dental qualification?

UK dental school

EU dental school

Non-EU dental school

Q6 Are you a registered specialist or undertaking specialty training?

No

Yes – I am a registered specialist

Yes - I am undertaking specialty training

Q7 In which NHS region did you mainly work since the start of the COVID-19 pandemic in March 2020?

East of England

London

Midlands

North-East & Yorkshire

North-West

South-East

South-West

Not sure

Q8 Which sector did you work in during 2020-2021? (please select all that apply)

General Dental Practice – NHS

General Dental Practice – Mixed

General Dental Practice – Private

Community Dental Service

Urgent Dental Centre – Remote and face to face dentistry

Urgent Dental Centre – Remote only

Other – please state

Q9 Did the COVID-19 pandemic affect the number of antibiotics you prescribed between April 2020 and March 2021, compared to the previous year (April 2019 to March 2020)? [PRIMARY RESEARCH QUESTION]

Many more antibiotics during the COVID-19 pandemic than previously

Slightly more antibiotics during the COVID-19 pandemic than previously

About the same number of antibiotics during the COVID-19 pandemic as previously

Slightly fewer antibiotics during the COVID-19 pandemic than previously

Far fewer antibiotics during the COVID-19 pandemic than previously

Q10 How clear did you find the national guidance on the remote management of patients (advice, analgesics and antimicrobials, where appropriate) issued on 25 March 2020?

Extremely clear

Somewhat clear

Neither clear nor unclear

Somewhat unclear

Extremely unclear

Q11 How well did local guidelines and practices align with national guidance on the remote management of patients (advice, analgesic and antimicrobials, where appropriate) issued on 25 March 2020?

Extremely well

Very well

Moderately well

Slightly well

Not well at all

Q12 Did you prescribe antibiotics remotely? [please select all that apply]

Yes during April – May 2020 (when general dental practices were closed)

Yes during June – July 2020 (when NHS dentistry had no minimum activity threshold)

Yes during August – December 2020 (when NHS dentistry had a 20% minimum activity

threshold)

Yes during January – March 2021 (when NHS dentistry had a 45% minimum activity threshold

Yes since April 2021 (when NHS had a 60% minimum activity threshold)

No

Don’t know

Q13 Have you been aware of antibiotics being prescribed remotely in your local NHS area? [please select all that apply]

Yes during April – May 2020 (when general dental practices were closed)

Yes during June – July 2020 (when NHS dentistry had no minimum activity threshold)

Yes during August – December 2020 (when NHS dentistry had a 20% minimum activity threshold)

Yes during January – March 2021 (when NHS dentistry had a 45% minimum activity threshold

Yes since April 2021 (when NHS had a 60% minimum activity threshold)

No

Don’t know

Q14 Were any of your referrals to an Urgent Dental Centre rejected because the patient had not first taken antibiotics? [please select all that apply]

Yes during April – May 2020 (when general dental practices were closed)

Yes during June – July 2020 (when NHS dentistry had no minimum activity threshold)

Yes during August – December 2020 (when NHS dentistry had a 20% minimum activity

threshold)

Yes during January – March 2021 (when NHS dentistry had a 45% minimum activity threshold

Yes since April 2021 (when NHS had a 60% minimum activity threshold)

No

Don’t know

Q15 Was there a time when you prescribed antibiotics to delay treatment until you could provide an aerosol-generating procedure? [please select all that apply]

Yes during April – May 2020 (when general dental practices were closed)

Yes during June – July 2020 (when NHS dentistry had no minimum activity threshold)

Yes during August – December 2020 (when NHS dentistry had a 20% minimum activity

threshold)

Yes during January – March 2021 (when NHS dentistry had a 45% minimum activity threshold

Yes since April 2021 (when NHS had a 60% minimum activity threshold)

No

Don’t know

Q16 How confident did you feel diagnosing acute dental pain or infection remotely?

Extremely confident

Somewhat confident

Neither confident nor unconfident

Somewhat unconfident

Extremely unconfident

Q17 How confident did you feel treating acute dental pain or infection remotely?

Extremely confident

Somewhat confident

Neither confident nor unconfident

Somewhat unconfident

Extremely unconfident

Q18 How often did patients request antibiotics during COVID (April 2020 to March 2021) compared to the previous year (April 2019 to March 2020)?

More in 2020-2021

Fewer in 2020-2021

Similar in each year

Don’t know

Q19 How many patients previously unknown to you did you provide care for during COVID (April 2020 to March 2021) compared to the previous year (April 2019 to March 2020?

More in 2020-2021

Fewer in 2020-2021

Similar in each year

Don't know

Q20 Is there anything else you would like to add, to help us better understand how COVID-19 impacted on dental antibiotic prescribing in England?
